# Intrapericardial diaphragmatic hernia into the pericardium after esophagectomy: a case report

**DOI:** 10.1186/s40792-018-0499-z

**Published:** 2018-08-13

**Authors:** Takuya Saito, Kohei Yasui, Shintaro Kurahashi, Kenichi Komaya, Seiji Ishiguro, Takashi Arikawa, Shunichiro Komatsu, Kenitiro Kaneko, Masahiko Miyachi, Tsuyoshi Sano

**Affiliations:** Division of Gastroenterological Surgery, Department of Surgery, Aichi Medical University, 1-1 Yazakokarimata, Nagakute, Aichi 480-1195 Japan

**Keywords:** Esophageal cancer, Intrapericardial hernia, Diaphragmatic hernia, Esophagectomy, Autologous graft, Rectus abdominis sheath

## Abstract

**Background:**

Intrapericardial diaphragmatic hernia (IPDH), defined as prolapse of the abdominal viscera into the pericardium, is a rare clinical condition. This case illustrates the possibility of IPDH after esophagectomy with antethoracic alimentary reconstruction, although such hernias are extremely rare. IPDH often presents with symptoms of bowel obstruction such as abdominal discomfort or vomiting. If not properly treated, life-threatening necrosis and/or perforation of the herniated contents may occur.

**Case presentation:**

A 68-year-old Japanese man underwent subtotal esophagectomy with three-field lymph node dissection for treatment of esophageal cancer. Completion gastrectomy with perigastric lymph node dissection was also performed because the patient had previously undergone distal partial gastrectomy for treatment of gastric cancer. The alimentary continuity was reconstructed using the pedicled jejunal limb through the antethoracic route. When we separated the diaphragm from the esophagus and removed xiphoid surgically to prevent a pedicled jejunal limb injury, the pericardium was opened. The patient was readmitted to our hospital because of abdominal discomfort and vomiting 6 months after the esophagectomy. A diagnosis of IPDH after esophagectomy was made. The patient was treated by primary closure of the diaphragmatic defect using vertical *mattress* sutures and additional reinforcement of the closing defect using a graft harvested from the rectus abdominis posterior sheath. The postoperative course was uneventful, and he was discharged on the seventh day after hernia repair.

**Conclusions:**

This patient’s clinical course provides two important clinical suggestions. First, we must be aware of the possibility of iatrogenic IPHD after esophagectomy with antethoracic alimentary reconstruction. Second, a graft from the rectus abdominis posterior sheath is beneficial in the treatment of IPDH.

## Background

Intrapericardial diaphragmatic hernia (IPDH) is a rare clinical condition. IPDH is defined as prolapse of the abdominal viscera from the peritoneal cavity into the pericardium. IPDH often presents with symptoms of bowel obstruction, such as abdominal discomfort or vomiting. If not properly treated, life-threatening necrosis and/or perforation of the herniated contents may occur. IPDH is often a congenital etiology, but it may also be caused by prior trauma or interventional and surgical procedures. Iatrogenic IPDH was first described by Swartz et al. in 1984 [1], who reported this condition as a complication after cardiac surgery. We experienced an extremely rare case of iatrogenic IPDH after esophagectomy. Although some cases of diaphragmatic hernia after esophagectomy have been reported, herniation into the pericardium as described in the present case has not been reported. This is the first reported case of IPDH after esophagectomy. The details of the surgical treatment for IPDH are not well established. We herein report a new treatment option to prevent recurrent IPDH using a graft harvested from the rectus abdominis posterior sheath.

## Case presentation

**Fig. 1:**
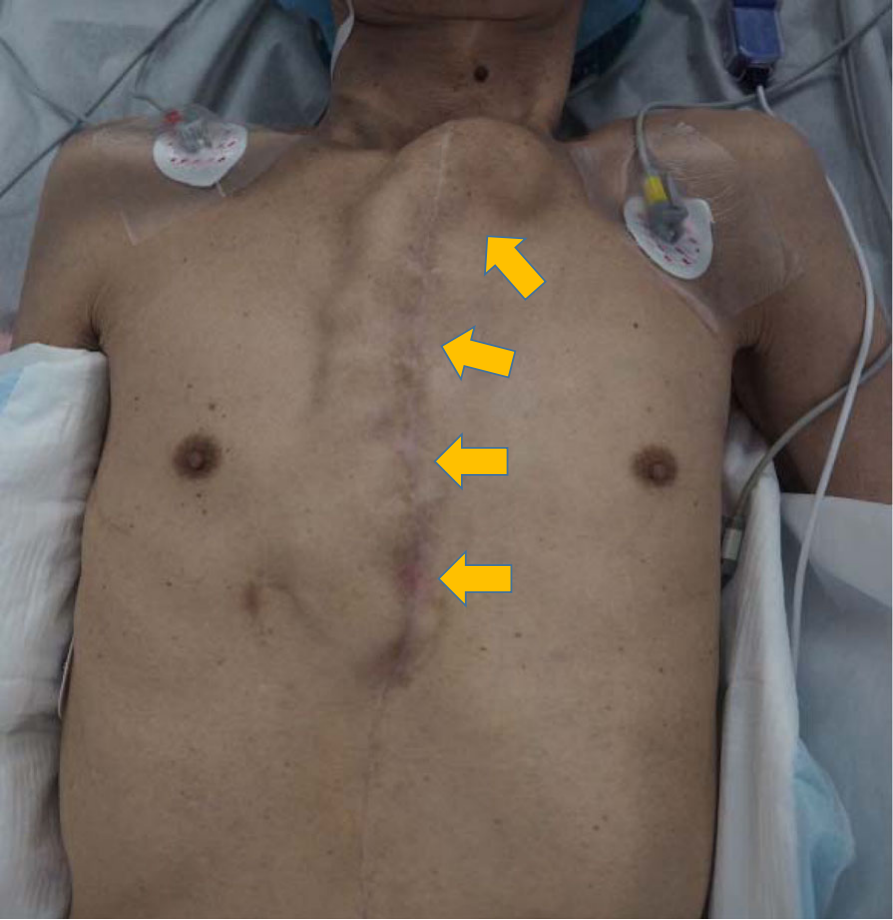
Photograph of the patient taken just prior to surgery shows the significantly dilated pedicled jejunum through the antethoracic route (arrows)

**Fig. 2:**
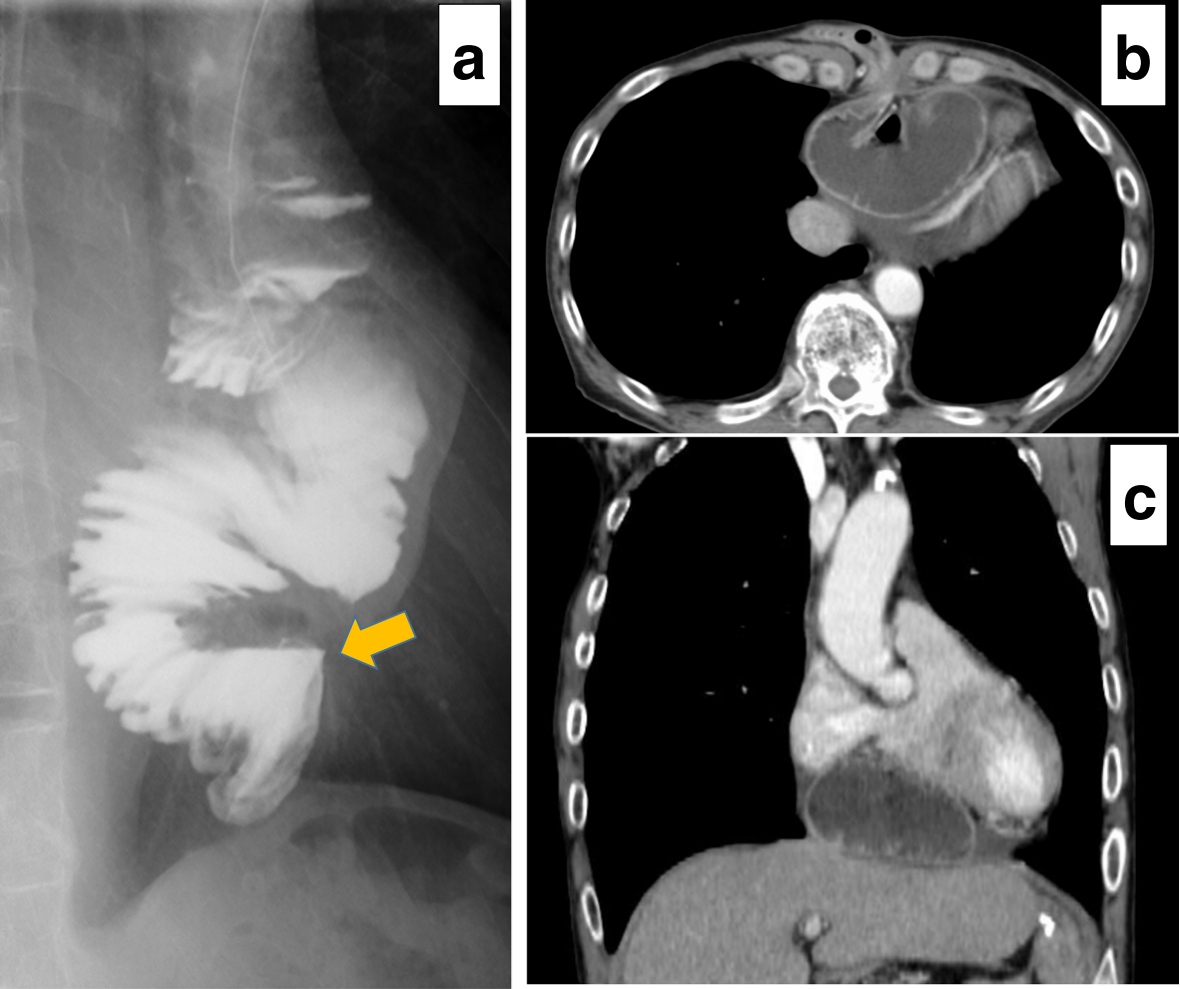
**a** The pedicled jejunum through the antethoracic route shows the beak sign (arrow) in the enteral contrast study. **b** Transverse and **c** sagittal enhanced computed tomography images demonstrate the intrapericardial migration of the small bowel anterior to the heart (arrows)

**Fig. 3:**
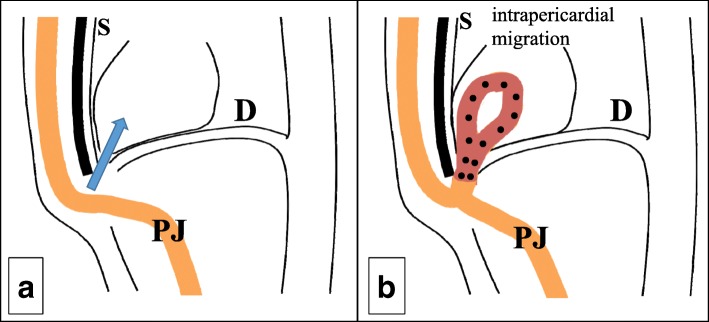
Schematic illustration of **a** the antethoracic reconstruction and **b** the completed herniation. *PJ*: pedicled jejunum, *D*: diaphragm, *S*: sternum

**Fig. 4:**
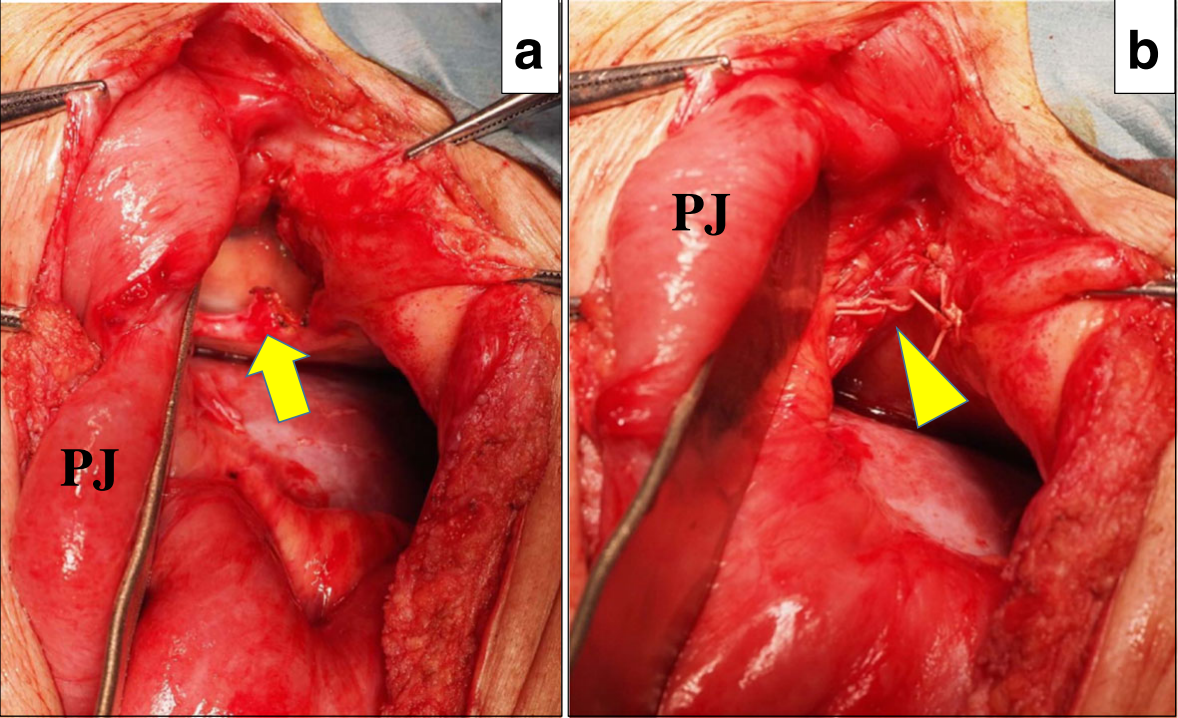
Intraoperative photographs show **a** the hernia orifice (arrow) and **b** the primary suture line. *PJ*: pedicled jejunum

**Fig. 5:**
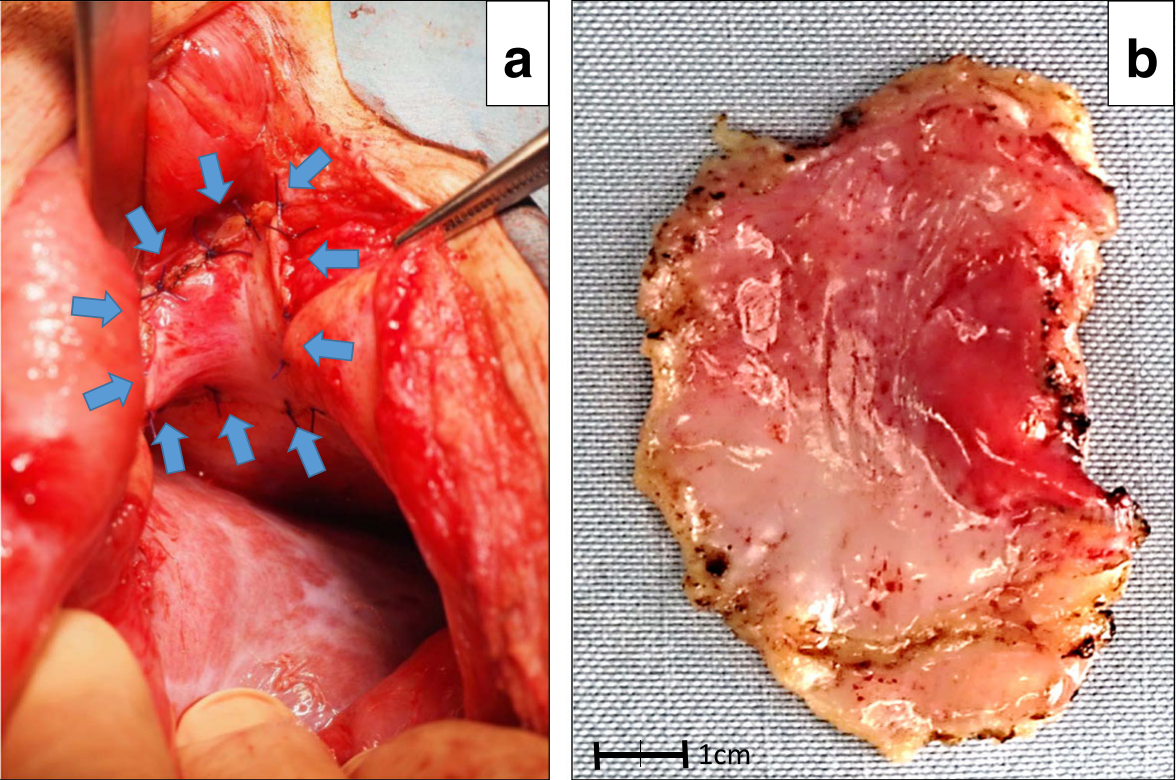
**a** The closed diaphragmatic defect was reinforced with a graft (8 × 6 cm) of the rectus abdominis posterior sheath. **b** This photograph shows the 8 × 6 cm graft harvested from the rectus abdominis posterior sheath and the reinforcement with the graft

A 68-year-old Japanese man with a history of distal partial gastrectomy for gastric cancer 10 years earlier was admitted for surgical treatment of intrathoracic esophageal cancer (T3, N2, M0, stage III). He underwent subtotal esophagectomy with three-field lymph node dissection and removal of the remnant stomach with the abdominal lymph nodes. The alimentary continuity was reconstructed with a pedicled jejunal limb through the antethoracic route. When we separated the diaphragm from the esophagus and removed xiphoid surgically to prevent a pedicled jejunal limb injury, the pericardium was opened. Anastomosis of the esophagus and jejunum was carried out instrumentally with a circular stapler. A postoperative enteral contrast examination showed smooth passage and no deformity of the reconstructed jejunum. The patient was discharged about 4 weeks postoperatively. About 6 months after the esophagectomy, the patient was readmitted to our hospital because of abdominal discomfort and vomiting. His vital signs were stable and unremarkable. He was thin, with a body mass index of 18.6 kg/m^2^. Physical examination revealed that the pedicled jejunum through the antethoracic space was markedly dilated and the abdomen was soft and flat (Fig. 1). Laboratory data showed only leukocytosis with no other remarkable findings. A chest roentgenogram revealed an increased cardiothoracic ratio of 70%, and an enteral contrast study showed a bird’s beak deformity. The swallowed barium remained static in the reconstructed jejunum. Computed tomography of the thoracoabdominal region showed the reconstructed jejunum within the pericardium anterior to the heart (Fig. 2). We diagnosed the patient with IPDH after esophagectomy and performed an emergency laparotomy. Upon opening the abdominal cavity, we found that the reconstructed jejunum had herniated into the pericardium through an approximately 4-cm-diameter diaphragmatic defect without a hernia sac (Fig. 3). The herniation was easily reduced, and the congestion of the incarcerated jejunum resolved. There was no evidence of bowel ischemia. Primary closure of the diaphragmatic defect was accomplished using vertical *mattress* sutures with 1–0 silk (Fig. 4). Because the diaphragm adhered to the pericardium around the diaphragmatic defect, we closed these together. Moreover, to reinforce the closure of the diaphragmatic defect, we used a graft harvested from the rectus abdominis posterior sheath. Interrupted sutures with 3–0 nylon were placed to fix the 8 × 6 cm graft of the rectus abdominis posterior sheath to the diaphragmatic defect, preventing recurrence of the hernia (Fig. 5). Postoperatively, an upper gastrointestinal study confirmed free flow of contrast medium from the cervical esophagus into the intra-abdominal jejunum through the pedicled jejunum. The postoperative course was uneventful, and the patient was discharged on the seventh postoperative day.

## Conclusions

IPDH, the rarest of all non-hiatal diaphragmatic hernias, is a sacless hernia [2, 3]. IPDH is a communication between the peritoneal cavity and pericardium, and a gap in the pericardial portion of the central tendon of the diaphragm is frequent route [4]. IPDH can have a traumatic, congenital, or iatrogenic etiology. Among these three etiologies, an iatrogenic cause is extremely rare [2]. Iatrogenic IPDHs have reportedly occurred after coronary artery bypass grafting using a right gastroepiploic artery graft [5] and subxiphoid epicardial pacemaker insertion through a pericardial-peritoneal window [6].

Although some cases of diaphragmatic hernia after esophagectomy have been reported, the hernia orifice in these cases was located at the hiatus only [7]. No reports have described the hernia orifice at the pericardium, as in the present case. Therefore, this is the first reported case of iatrogenic IPDH after esophagectomy. The retrosternal or mediastinal route is usually chosen for the alimentary reconstruction after esophagectomy. IPDH has not been reported as a complication after esophagectomy because surgeons rarely choose to perform the reconstruction by the antethoracic route. Surgeons must consider the various types of diaphragmatic hernias that may occur according to the reconstruction route after esophagectomy.

A possible mechanism of jejunal limb herniation into the pericardium in the present case is the opening of the pericardium during the esophagectomy with antethoracic alimentary reconstruction. Because the xiphoid and pericardium are anatomically adjacent, the pericardium was opened during xiphoidectomy. IPDH occurred by jejunal peristalsis and pressure overload. It is important to prevent the pericardium from opening during xiphoidectomy. When the pericardium is opened, surgeons should tightly close it.

We have herein reported a new treatment option to prevent recurrent intrapericardial herniation using a graft harvested from the rectus abdominis posterior sheath. Both primary suture plication to close the defect and reinforcement of the suture closure are crucial for effective hernia treatment. The details of the surgical treatment for IPDH are not yet established. Kovacich et al. [8] reported that the principles of the operation are reduction of the herniated organs, definition of the edges of the diaphragmatic hernia, and closure of the defect. Several reports have described reinforcement of the diaphragmatic incision with prosthetic mesh [9, 10]. The true rate of mesh-related complications associated with reinforcement of the hernia orifice is still unknown. We were concerned about complications related to mesh attachment to the pedicled jejunum. To prevent recurrence, we closed the diaphragmatic defect and reinforced it with a graft from the rectus abdominis posterior sheath. Two case reports have described repair of IPDH with an autologous graft [11]. The falciform ligament or left triangular ligament was used as the autologous graft. These case reports noted that the surgical procedure was simple, easy, and safe. Because both ligaments were separated during the esophagectomy, one may not use it; however, reinforcement with a graft of the rectus abdominis posterior sheath was useful. The rectus abdominis posterior sheath is easily taken from the same operative field and is associated with a low rate of septic complications. Yigit et al. [12] reported that the rectus abdominis fascial sheath was useful for crural reinforcement of a hiatal hernia. Such a graft is more effective than mesh from the viewpoint of cost-effectiveness and infectious complications.

Our patient’s clinical course provides two important clinical suggestions. First, iatrogenic IPDH can occur in a patient who has undergone esophagectomy with antethoracic pedicled jejunal limb reconstruction. Second, a graft harvested from the rectus abdominis posterior sheath is useful for repair and/or reinforcement of IPDH.

## Abbreviations

IPDH: Intrapericardial diaphragmatic hernia
